# Smart three-dimensional lightweight structure triggered from a thin composite sheet via 3D printing technique

**DOI:** 10.1038/srep22431

**Published:** 2016-02-29

**Authors:** Quan Zhang, Kai Zhang, Gengkai Hu

**Affiliations:** 1 School of Aerospace Engineering, Beijing Institute of Technology, Beijing 100081, China

## Abstract

Complex fabrication process and expensive materials have restricted the development of smart three-dimensional (3D) lightweight structures, which are expected to possess self-shaping, self-folding and self-unfolding performances. Here we present a simple approach to fabricate smart lightweight structures by triggering shape transformation from thin printed composite sheets. The release of the internal strain in printed polymer materials enables the printed composite sheet to keep flat under heating and transform into a designed 3D configuration when cooled down to room temperature. The 3D lightweight structure can be switched between flat and 3D configuration under appropriate thermal stimuli. Our work exploits uniform internal strain in printed materials as a controllable tool to fabricate smart 3D lightweight structures, opening an avenue for possible applications in engineering fields.

Smart three-dimensional (3D) lightweight structures are expected to possess self-shaping, self-folding and self-unfolding functions, which may have broad applications in assembly^1^,^2^,^3^,^4^,^5^,^6^, packaging^7^, robot actuator^8^,^9^,^10^, solar cells^11^, drug delivery and biological devices^12^,^13^,^14^. Complex fabrication process and expensive materials have restricted their development. A potential approach to fabricate such lightweight structure is the shape transformation from a flat sheet under external stimuli^15^,^16^. A typical mechanism of the approach involves non-uniform internal stress, generated via contrasted materials under swelling or heating^17^,^18^. Such formed structures can achieve a reversible unfolding deformation, compared with traditional technique by controlling local recovery of shape memory polymers^19^. Nowadays, novel structures with customized materials can be fabricated rapidly by 3D printing technique^20^,^21^,^22^. However, the 3D printing technique is still challenged as a practical means to fabricate smart 3D lightweight structures with spontaneous responses under external stimuli.

It is reported that uniform internal strain is stored in printed polymer and it can be released when reheated, resulting in a heat-shrinkable phenomena of polymer materials^23^. Here we exploit the internal strain in printed polymer to fabricate smart 3D lightweight structures by self-folding thin sheets under thermal stimuli. The method integrates the advantages of 3D printing technique and the printed polymer with shape memory effect, thereby providing an efficient way to obtain complex 3D lightweight structures with self-shaping, self-folding/unfolding performances. This work opens a door not only to address novel mechanism on thermal-induced shape transformation of 3D-printed composite materials, but also to practical fabrication of smart 3D lightweight structures or devices.

## Results

### Three-dimensional lightweight structures triggered from printed composite sheets

**Figure 1:**
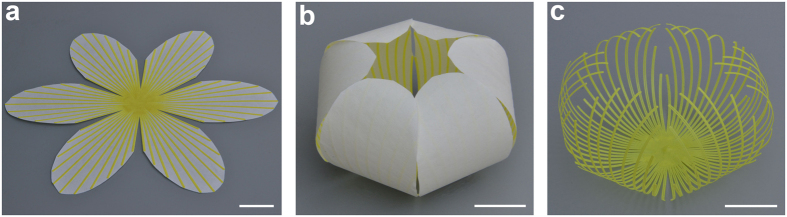
Formation of flower-like 3D structure from initial planar sheet. (**a**) The initial planar shape of the 3D-printed composite sheet. (**b**) The final flower-like 3D shape after a process of heating and cooling. (**c**) A complex lightweight structure fabricated by tearing off the paper from the flower-like 3D structure. Scale bar is 2 cm.

We fix a membrane of paper sheet, on the platform of a 3D printer. As shown in Fig. 1a, flat polylactic acid (PLA) strips with the width of 0.8 mm and thickness of 0.2 mm, which are similar to the structure of leaf vein, are printed on the fixed sheet. Then we cut the composite sheet to a shape with six petals and put it on a heating plate at 105 °C, keeping the layer of PLA materials faced up. It is common that multi-layer membrane structure, consisting of different materials in its thickness, can generate a 3D structure spontaneously under heating and recover to its initial flat shape gradually after cooling due to the mismatching coefficient of thermal expansion (CTE) between different materials. As expected, the center of the 3D-printed composite sheet bends upward in the beginning, since the CTE of PLA materials on the top layer is larger than that of paper. However, as the temperature increases, the whole composite sheet bends toward the opposite direction spontaneously, even making the composite sheet remain almost flat after reaching equilibrium temperature (Supplementary Video 1). The whole composite sheet can form a 3D structure with lifted edge at equilibrium state of heating process when the thickness of the printed PLA is increased. As the composite sheet is cooled down, the planar composite sheet will be transformed into a flower-like 3D shape spontaneously (Fig. 1b and Supplementary Video 2). The newly formed 3D structure can recover its initial planar shape when reheated (Supplementary Video 3), demonstrating that the processes of self-folding and self-unfolding are completely reversible. Thus, the 3D-printed composite sheet possesses the characteristics of self-shaping, reversible self-folding and self-unfolding. By tearing off the paper from the flower-like 3D structure, a complex lightweight structure is also obtained in Fig. 1c, which is difficult to fabricate by 3D printing technique.

**Figure 2:**
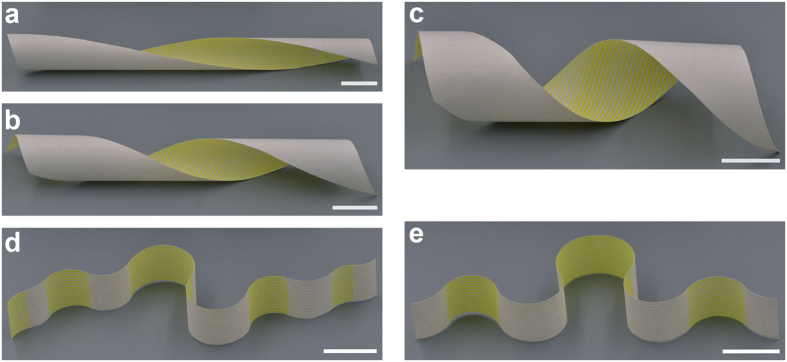
Different kinds of 3D structures created by self-folding printed composite sheets. (**a–c**) Helical structures with different degrees of spiral. (**d,e**) Corrugated structures by bidirectional folding of printed composite sheets. Scale bar is 2 cm.

**Figure 3:**
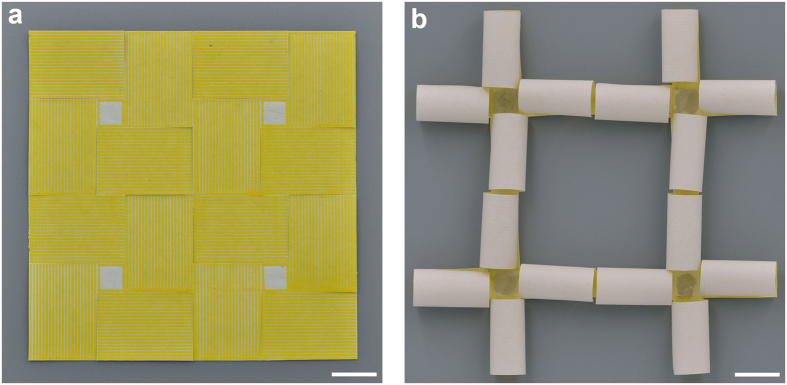
Formation of the periodic 3D lightweight structure from printed composite sheet. (**a**) Initial periodic printed composite sheet. (**b**) Final orthogonal periodic structure after a cycle of heating and cooling. Scale bar is 2 cm.

Similarly, a number of smart 3D lightweight structures can be triggered from the planar composite sheets under thermal stimuli. Figure 2a–c shows some examples of constructing helical structures with different helical angles. After a piece of paper with width of 3 cm is fixed on the platform, PLA strips are printed on the paper with an angle *β*, defined as the angle between printed strips and horizontal direction. The width and thickness of the PLA strips are 0.6 mm and 0.2 mm, respectively, and the space between adjacent strips is 0.9 mm. *β* corresponding to Fig. 2a–c is π/3, π/4 and π/6, respectively. Similar to the fabricating process of the flower-like 3D structure, after being heated on a heating plate at 105 °C and then cooled down to room temperature, thin flat composite sheets roll up to form the helix structures. By printing staggered PLA strips on both surfaces of a rectangular paper, corrugated structures can also be formed under the similar cycle of heating and cooling, as show in Fig. 2d,e. More interestingly, periodic 3D lightweight structure can also be formed by printing PLA strips periodically on a flat paper sheet. As shown in Fig. 3a, a composite sheet, consisting of four periodic cells in square arrangement, is fabricated. Each cell includes a central region without PLA strips and four rectangular regions with printed PLA strips. The sizes of the cell and the central region are 7.2 × 7.2 cm and 1.2 × 1.2 cm, respectively. Orthogonal PLA strips are printed on neighbor rectangular regions and the public edges between them are cut off. The width and thickness of the PLA strips are 0.8 mm and 0.2 mm, respectively, and the space between adjacent strips is 0.6 mm. After undergoing the similar process of heating and cooling, a periodic 3D lightweight structure is triggered (shown in Fig. 3b). All of the newly formed 3D structures above can recover their original planar shapes when reheated.

### Thermal response of PLA materials printed on paper sheet

**Figure 4:**
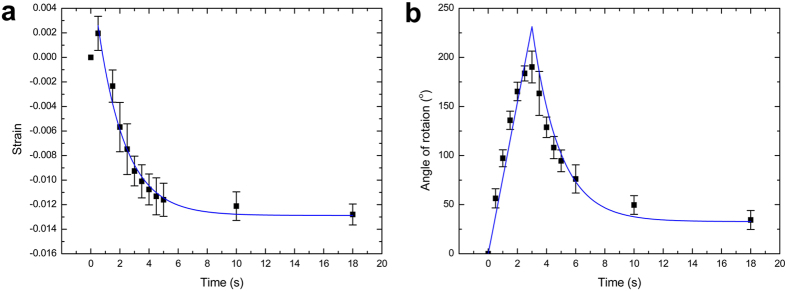
(**a**) Experimental data and theoretical strain-time curve for printed PLA strips. (**b**) Experimental and theoretical angle of rotation for printed composite strips.

In our previous work, we have explained how internal strain is generated when the PLA materials is printed directly on the platform of 3D printer^23^. Here we firstly evaluate internal strain stored in PLA materials when it is printed on a fixed paper. Long PLA strips with the size of 90 × 1.6 × 0.12 mm (length × width × thickness) are printed on a piece of fixed paper. Then we tear off the paper and put the long PLA strips on the surface of a heating plate at 90 °C. By calculating the ratio of the contraction to the initial length of the strips, we obtain the strains of the long strips during the deformation process, as shown in Fig. 4a. As can be seen that the printed PLA strips expand in the beginning, but the expansion process is very short because the strips are so thin that they reach the equilibrium temperature very quickly. Subsequently, the printed PLA strips present a contraction phenomenon, resulting from the release of internal strain when their temperature exceeds the glass transition temperature of PLA (*T*_*g*_, 60 °C)^24^,^25^. We consider the moment 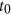 corresponding to *T*_*g*_ as the beginning of the releasing process, a classic Voigt model which consists of a spring and a dashpot can be used to describe the strain of printed PLA strip 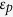 with increasing time (*t*)^23^:


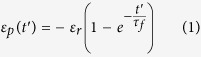


Where 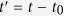. We can obtain the stored internal strain 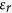 0.0155 and the relaxation time 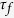 1.89 s, respectively, by using equation (1) to fit the experimental data in Fig. 4a. By defining an equivalent linear CTE 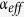 through 
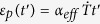
 with heating rate 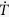, the equivalent linear CTE of the printed materials for the releasing process of internal strain is given as


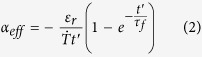


In comparison with common materials with constant CTE, when the 3D-printed materials is reheated, it behaves as common PLA materials in the beginning, but shows an equivalent negative linear CTE as soon as its temperature is above *T*_*g*_. After the residual strain has been released completely, the equivalent linear CTE approaches to zero.

### Bending deformation of printed composite strip under thermal stimuli

Now we explain the deformation of printed composite strips under heating. Similarly, long composite strips, which consist of paper and PLA strip with the size of 60 × 0.8 × 0.12 mm (length × width × thickness), are fabricated. The lateral side of the composite strip is heated on the heating plate at 90 °C and the angle *θ* between the normals of both ends of the composite strip is measured. As shown in Fig. 4b, the angle increases with time in the beginning, indicating that the composite strip starts to bend toward the direction of paper under heating due to the contrasted CTE between the printed polymer and paper. According to the reference^26^, when the temperature is below *T*_*g*_, the bending angle for the thermal expansion process can be given as


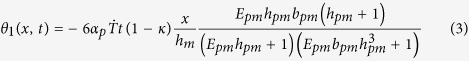


Where 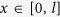 and *l* means the length of the composite strip, 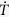 is the heating rate. *p* and *m* denote the printed polymer and membrane of paper, respectively; 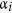, *h*_*i*_ , *b*_*i*_ are the CTE, thickness and width of the materials (*i* = *p*, *m*), respectively; 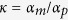, 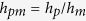, 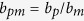. 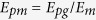, *E*_*pg*_ and *E*_*m*_ denote the elastic modulus of the printed polymer and paper below *T*_*g*_, respectively.

After heating the composite strip for about 3 s, the angle *θ* drops sharply and then keeps constant (Fig. 4b). The release of internal strain stored in PLA strip can generate a negative equivalent linear CTE of 3D-printed materials, and further result in an obvious bending of the composite sheet in the opposite direction of bending deformation, which enables the printed composite sheet to keep in planar state. For heating process over *T*_*g*_ , the corresponding bending angle is expressed as:


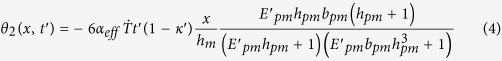


Where 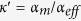, 
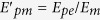
, *E*_*pe*_ is the elastic modulus of the printed polymer over *T*_*g*_. Therefore, the total bending angle for 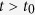 will be expressed as the sum of 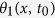 and 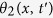, where 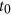 represents the moment at which temperature of the composite strip reaches *T*_*g*_. Finally, the theoretical result is plotted in Fig. 4b, showing fine agreement with the experimental result (Parameters of the composite strip seen in Methods).

**Figure 5:**
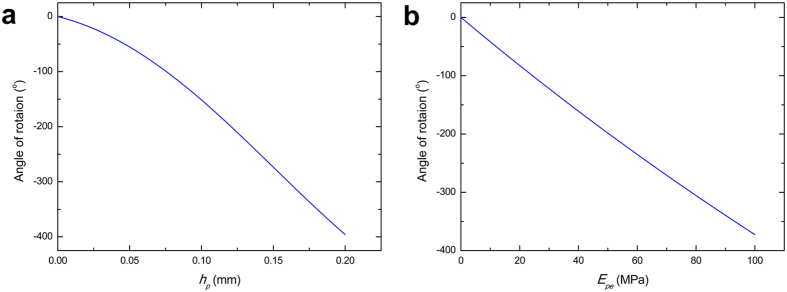
The relationship between the final bending angle of the composite strip and the thickness (**a**) or elastic modulus over *T*_*g*_ of printed PLA strip (**b**).

After the printed composite strip reaches its equilibrium state on heating plate, removing it from the heating plate makes the printed composite strip bend toward the direction of PLA materials. As a result, a curved strip is formed spontaneously. The final shape of the curved strip is described by Equation (4), that is, the shape transformation is only dependent on the released internal strain, having no relations to the interaction between contrasted CTE of materials. We plot the final bending angle of the composite strip with varying thickness (*h*_*p*_) and elastic modulus over *T*_*g*_ (*E*_*pe*_) of printed PLA in Fig. 5. It can be seen that appropriately increasing *h*_*p*_ or *E*_*pe*_ of printed materials finally forms the curved composite strip with larger bending angle. Since the formation of the final curved strip is due to the thermal elastic deformation generated during cooling process, the curved strip can recover the initial flat strip reversibly under reheating. For the printed composite sheet, it can be regarded as the combination of a series of composite strips. The total bending moment is the sum of the bending moment generated from each composite strip (expressed in Supplementary Note), then the bending angle is further obtained to describe the deformation of the printed composite sheet.

Theoretically, the cycle times of folding and unfolding is infinite since the formation of the final 3D structure is due to the thermal elastic deformation generated during cooling process. However, according to our experimental observation, after undergoing a dozen times of heating and cooling cycle, the final 3D structure can’t recover to the initial flat sheet completely under reheating due to the aging of polymer materials. In addition, the internal strain stored in PLA materials is relatively small (about 0.0155 with a building speed of 30 mm s^−1^), when it is printed on a fixed paper. According to our previous work^23^, increasing both the building speed and the elastic modulus of the substrate significantly result in larger internal strain stored in printed PLA materials. Thus the self-folding/unfolding force of these smart 3D lightweight structures can be enhanced by using hard paper board as well as increasing the building speed.

### The proposed adaptive metamaterials

Conventional composite sheet consisting of different materials can also lead to self-folding. However, the 3D configuration can only be kept at abnormal temperature. In comparison, the printed composite sheet can be triggered into 3D configuration at room temperature but transforms into the planar state in hot environment. Furthermore, it is reported that the 3D printing technique generates a uniform internal strain in polymer in a controllable way^23^, enabling the shape of the printed composite membrane to be transformed precisely. Our method may inspire the innovation of 3D printing technique to fabricate smart lightweight structures or devices for specific applications, such as mechanical metamaterials, optical components and tissue engineering.

**Figure 6:**
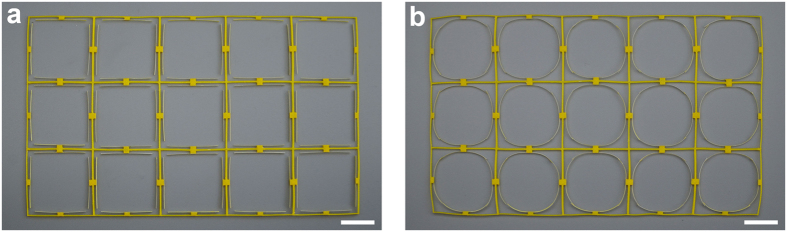
Fabricated adaptive metamaterials. (**a**) The initial structure is constructed by introducing auxiliary beams into standard two-dimensional square lattice materials. (**b**) After undergoing a process of heating and cooling, the whole lattice structure transforms into a new configuration with curved beams. Scale bar is 2 cm.

**Figure 7:**
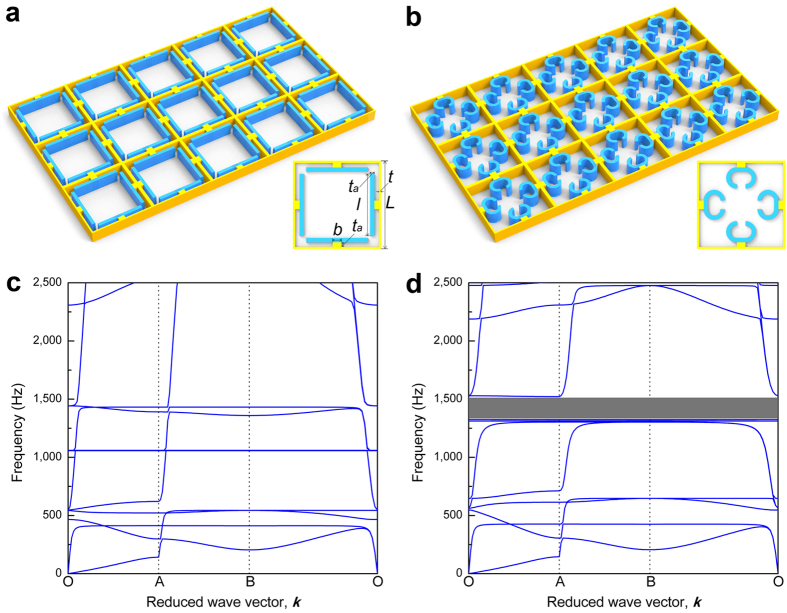
Proposed adaptive metamaterials and corresponding band structures. (**a**) The initial configuration with straight beams. (**b**) The configuration with curved beams after undergoing a process of heating and cooling. (**c**) A clear band gap exists when the auxiliary composite beams in the lattice are in curve configuration and (**d**) disappears once the auxiliary composite beams deform to be straight under heating.

For example, inspired by the self-folding mechanism we reported, here we conceptually propose a new type of adaptive metamaterials, whose band gaps can be switched by reversibly changing the configurations of lattice structures under thermal excitation. Although lattice structures have attracted much attention in recent years mainly for their abilities to manipulate and control elastic waves^27^,^28^, most lattice structures can only have band gaps with fixed frequency ranges or even have no band gaps, limiting more potential applications. It is reported that mechanical instability^29^,^30^, fluid-structure interaction^31^, and piezoshunting^32^,^33^ can be employed to achieve adaptive metamaterials with tunable bandgaps. Here the proposed adaptive metamaterials is constructed by introducing self-folding beams into standard two-dimensional square lattice materials, as shown in Fig. 6a. Composite beam, fabricated by printing PLA on a membrane, is attached to each wall of a lattice structure. According to the self-folding mechanism, after undergoing a process of heating and cooling, the attached straight beams are folded to curved beams, thus the whole lattice structure transforms into a new configuration as depicted in Fig. 6b. Afterwards, the lattice structure can switch back and forth between these two configurations under appropriate thermal stimuli. The schematic diagrams and band structures for this kind of adaptive metamaterials are shown in Fig. 7.

We use commercial software COMSOL Multiphysics to simulate the band structures corresponding to these two configurations in Fig. 7a,b. In the simulation, we focus on a representative element, which comprises a square main frame, four composite beams fabricated by printing PLA on membranes, and four rectangular solids which connect the composite beams to the main frame. The main frame along with all the rectangular solids are made of PLA materials, the composite beams are equivalent to uniform beams with the same material parameters as PLA. In the simulation, plain strain triangular element is chosen with the maximum element size of 3.0*10^−4^ m. The band structure diagrams are presented in Fig. 7c,d and there is a clear band gap for the configuration with curved beams (Fig. 7d). Several flat bands at frequency about 1,311 Hz, which corresponds to the first natural frequency of a resonant structure consisting of the curved beam and the rectangular solid, indicate a typical feature of band gap based on local resonance. However, the band gap related to local resonance will completely close when the lattice structure transformed into the other configuration (Fig. 7c). The proposed metamaterials can be therefore used as a wave switch that provides on or off function.

## Conclusion

We investigate the thermal response of polymer materials printed on a paper sheet and present a novel approach for fabricating smart 3D lightweight structures by printing PLA strips on a membrane. The corresponding mechanism is that the release of the uniform internal stress in 3D-printed materials, generated during 3D printing process, counteracts the deformation caused by the mismatching CTE of composite materials. As a result, the printed composite sheet remains in flat state on heating plate, but transforms into 3D configuration when cooled down to room temperature. The 3D structure can recover its initial flat shape reversibly under heating, since the formation of the final 3D structure is due to the thermal elastic deformation during the cooling process. Finally, inspired by the self-folding mechanism we reported, we conceptually propose a type of adaptive metamaterials constructed by introducing self-folding beams into standard two-dimensional square lattice materials. The simulation results indicate that the proposed metamaterials can be used as a wave switch in a particular frequency range. Our study offers new insights into the design, manufacture and application of smart lightweight structures in engineering fields.

## Methods

The printed material in our work is PLA Filament, whose glass transition temperature (*T*_*g*_) is about 60~65 °C. Mechanical properties of PLA including tensile modulus, tensile strength, and thermal analysis by using a differential scanning calorimetry (DSC) and dynamic mechanical analyses (DMA) can be found in other literatures^25^.

All the composite sheets are fabricated by printing PLA materials on fixed paper sheets via a 3D polymer printer (MakerBot Replicator 2, MakerBot, Brooklyn, NY 11201 USA) with the highest resolution 0.1 mm per layer. The nozzle temperature is fixed to be 230 °C and the building speed is set to be 90 mm s^−1^. The ambient temperature is about 20 °C.

Long PLA strips with the size of 90 × 1.6 × 0.12 mm (length × height × thickness) are printed on fixed paper with a resolution of 0.12 mm per layer. The building speed is 30 mm s^−1^ and the nozzle temperature is fixed to be 230 °C. Afterwards, we tear off the paper and put the PLA strips on a heating plate with temperature of 90 °C. The deformation process of the printed PLA strips under heating is recorded using a digital camera. Then we measure the contraction of the long PLA strips at different moments and calculate the ratio of the contraction to the initial length of the strips. Five measurements have been made to obtain the mean strain with heating time.

To measure bending deformation of the printed composite materials, the size of the PLA strips is changed to be 60 × 0.8 × 0.12 mm (length × width × thickness), keeping the other printing conditions the same. The whole printed composite strips are then heated on a heating plate with temperature of 90 °C, the angle *θ* between the normals of both ends of the composite strip is also measured by digital camera. Five printed composite strips have been tested under heating and the mean bending angle with heating time is shown in Fig. 4b. In the theoretical calculation, the dimension of paper strip is 60 × 0.8 × 0.07 mm (length × width × thickness). The coefficient of thermal expansion of printed PLA is averaged to be 6.0×10^−5^/K by using a dilatometer (NETZSCH DIL 402 PC) and elastic modulus of paper is 5 GPa by using a testing machine (MTS, Eden Prairie, MN, USA). Five measurements are made to obtain the mean value, respectively. Elastic modulus of printed PLA is 3.5 GPa in the glass state according to the reference^24^,^25^ and assumed to be 50 MPa when the temperature is over its glass transition temperature (60 °C). The coefficient of thermal expansion of paper is assumed to be zero.

The representative element used in numerical simulation comprises (i) a square main frame with side length *L* = 40.0 mm and thickness *t* = 0.4 mm, (ii) four composite beams with length *l* = 31.5 mm and thickness *t*_*a*_ = 0.8 mm, fabricated by printing PLA on membranes, and (iii) four rectangular solids with the length *b* = 3.2 mm and thickness *t*_*a*_ which connect the composite beams to the main frame. The main frame along with all the rectangular solids are made of PLA materials, whose Young’s modulus *E* = 3.5 GPa, density *ρ* = 1270 kg m^−3^ and Poisson’s ratio *v* is assumed to be 0.33^24^,^25^.

## Additional Information

**How to cite this article**: Zhang, Q. *et al.* Smart three-dimensional lightweight structure triggered from a thin composite sheet via 3D printing technique. *Sci. Rep.* **6**, 22431; doi: 10.1038/srep22431 (2016).

## Supplementary Material

Supplementary Information

Supplementary Video 1

Supplementary Video 2

Supplementary Video 3
