# Supplementary material for: Smart three-dimensional lightweight structure triggered from a thin composite sheet via 3D printing technique
Source: Sci Rep. 2016 Feb 29;6:22431. doi: 10.1038/srep22431 (PMC4772624; doi:10.1038/srep22431)
Supplement: Supplementary Information [file srep22431-s1.doc]

**Supplementary Note**

**Smart three-dimensional lightweight structure triggered from a thin composite sheet via 3D printing technique**

Quan Zhang, Kai Zhang* and Gengkai Hu

School of Aerospace Engineering, Beijing Institute of Technology, Beijing 100081, China

E-mail: zhangkai@bit.edu.cn

**Theoretical analysis for the deformation of the printed composite strip under heating**

When the printed composite strip is put on a heating plate, it will be heated from room temperature (*T_s_*) to glass transition temperature (*T_g_*), until keeping the temperature of heating plate. As the printed polymer and paper is such thin that the stress field in them can be considered uniform. When the temperature is below *T_g_*, which means (denotes the heating rate), the composite strip expands under heating and the thermal stress in PLA due to the mismatching coefficient of thermal expansion (CTE) between the printed polymer and membrane of paper is given as^1^:

⑴

$\kappa=\alpha_{m}/\alpha_{p}$, $\gamma_{pm}=E_{pg}h_{p}/(E_{m}h_{m})$, $\alpha_{p}$ and $\alpha_{m}$, *h_p_* and *h_m_* are CTE and the thickness of the printed polymer and paper, respectively, *E_pg_* and *E_m_* denote the elastic modulus of the printed polymer and paper below *T_g_*, respectively. As the thermal stress is simplified to be uniform along the thickness of the composite strip, the bending moment then is calculated as^1^:

$M_{1}(t)=\sigma_{p1}(t)h_{p}(\frac{h_{p}+h_{m}}{2})b_{p}$ ⑵

Where *b_p_* is the width of the printed polymer. Thus, the composite strip is bent under the bending moment and the bending angle along the length of the strip is given as^1^:

$\theta_{1}\left(x,t\right)=\frac{M_{1}\left(t\right)x}{E_{pg}I_{p}+E_{m}I_{m}}$ ⑶

$x\in[0,l]$ and *l* means the length of the composite strip. Combine equations (1)-(3) we can obtain the expression of the bending angle for heating process below *T_g_*:

⑷

Where$E_{pm}=E_{pg}/E_{m}$, $h_{pm}=h_{p}/h_{m}$, $b_{pm}=b_{p}/b_{m}$, *b_m_* is the width of paper strip.

**References**

1 Timoshenko, S. & Gere J. M. *Mechanics of Materials* (Van Nostrand Reinhold Co., New York, 1973).
